# Plasma concentrations of glial fibrillary acidic protein, neurofilament light, and tau in Alexander disease

**DOI:** 10.1007/s10072-024-07495-8

**Published:** 2024-04-01

**Authors:** Nicholas J. Ashton, Di Molfetta Guglielmo, Kübra Tan, Kaj Blennow, Henrik Zetterberg, Albee Messing

**Affiliations:** 1 Department of Psychiatry and Neurochemistry, Institute of Neuroscience & Physiology, The Sahlgrenska Academy at the University of Gothenburg, Mölndal, Sweden; 2 Institute of Psychiatry, Psychology and Neuroscience, Maurice Wohl Institute Clinical Neuroscience Institute, King’s College London, London, UK; 3 NIHR Biomedical Research Centre for Mental Health and Biomedical Research Unit for Dementia at South London and Maudsley NHS Foundation, London, UK; 4 Centre for Age-Related Medicine, Stavanger University Hospital, Stavanger, Norway; 5 Clinical Neurochemistry Laboratory, Sahlgrenska University Hospital, Mölndal, Sweden; 6 Paris Brain Institute, ICM, Pitié-Salpêtrière Hospital, Sorbonne University, Paris, France; 7 Neurodegenerative Disorder Research Center, Division of Life Sciences and Medicine, and Department of Neurology, Institute On Aging and Brain Disorders, University of Science and Technology of China and First Affiliated Hospital of USTC, Hefei, People’s Republic of China; 8 Department of Neurodegenerative Disease, UCL Institute of Neurology, Queen Square, London, UK; 9 UK Dementia Research Institute at UCL, London, UK; 10 Hong Kong Center for Neurodegenerative Diseases, Clear Water Bay, Hong Kong, China; 11 Wisconsin Alzheimer’s Disease Research Center, University of Wisconsin School of Medicine and Public Health, University of Wisconsin-Madison, Madison, WI, USA; 12 Waisman Center, University of Wisconsin-Madison, Madison, WI 53705, USA; 13 Department of Comparative Biosciences, School of Veterinary Medicine, University of Wisconsin-Madison, Madison, WI 53705, USA

**Keywords:** GFAP, Alexander disease, Tau, Neurofilaments, Leukodystrophy, Plasma

## Abstract

**Introduction:**

Alexander disease (AxD) is a rare leukodystrophy caused by dominant gain-of-function mutations in the gene encoding the astrocyte intermediate filament, glial fibrillary acidic protein (GFAP). However, there is an urgent need for biomarkers to assist in monitoring not only the progression of disease but also the response to treatment. GFAP is the obvious candidate for such a biomarker, as it is measurable in body fluids that are readily accessible for biopsy, namely cerebrospinal fluid and blood. However, in the case of ASOs, the treatment that is furthest in development, GFAP is the target of therapy and presumably would go down independent of disease status. Hence, there is a critical need for biomarkers that are not directly affected by the treatment strategy.

**Methods:**

We explored the potential utility of biomarkers currently being studied in other neurodegenerative diseases and injuries, specifically neurofilament light protein (NfL), phosphorylated forms of tau, and amyloid-β peptides (Aβ42/40).

**Results and Conclusions:**

Here, we report that GFAP is elevated in plasma of all age groups afflicted by AxD, including those with adult onset. NfL and p-tau are also elevated, but to a much lesser extent than GFAP. In contrast, the levels of Aß40 and Aß42 are not altered in AxD.

## Introduction

Alexander disease (AxD) is a rare leukodystrophy caused by dominant gain-of-function mutations in the gene encoding the astrocyte intermediate filament, glial fibrillary acidic protein (GFAP) [13]. In all cases, the hallmark neuropathological feature is the formation of cytoplasmic protein aggregates in astrocytes known as Rosenthal fibres. A critical stage in the development of the disease seems to involve elevated levels of GFAP protein. This elevation is, in part, attributed to a positive feedback loop governing the regulation of GFAP expression. In this loop, the presence of the mutant protein triggers a stress response, leading to the transactivation of the *GFAP* promoter [8]. Studies using rodent models demonstrate that the suppression of GFAP expression, using antisense oligonucleotides (ASO), can not only prevent but even reverse the disease [5]. Based on these findings, a *GFAP*-targeted ASO has now moved into a combined phase 1–3 human clinical trial (ClinicalTrials.gov Identifier: NCT04849741).

Despite being a single gene disorder, more than 100 *GFAP* variants have been associated with AxD, with little genotype–phenotype correlation. A wide range of clinical features have been observed, with ages of onset spanning prenatal through the ninth decades, and symptomatology reflecting forebrain, hindbrain, and/or spinal cord dysfunction along with considerable variation in life expectancy [15, 19]. Nevertheless, the prospect of ASO and other potential therapies highlights an urgent need for quantitative biomarkers to assist in monitoring not only the progression of disease but also the response to treatment. GFAP is the obvious candidate for such a biomarker, as it is measurable in body fluids that are readily accessible for biopsy, namely cerebrospinal fluid and blood. Previously, we found that GFAP is markedly elevated in the cerebrospinal fluid of individuals with AxD and in blood of those with infantile and juvenile onset of symptoms (using a classification system based on age of first symptom) [7]. GFAP levels appeared unchanged in the blood of those with adult-onset disease. However, in the case of ASOs, the treatment that is furthest in development, GFAP is the target of therapy and presumably would go down independent of disease status. Hence, there is a critical need for biomarkers that are not directly affected by the treatment strategy.

Other biomarkers for neurodegenerative injury and pathologies have been identified in blood in recent years [6]. Neurofilament light (NfL) protein is primarily a biomarker reflecting degeneration of myelinated axons and is regarded as a measure of the intensity of ongoing injury and stage of neurodegeneration [11]. Both CSF and blood levels of NfL are increased in most neurodegenerative and acute neurological disorders. In addition, phosphorylated tau (p-tau) and amyloid-β peptides (Aβ42/40) in blood have been shown to be important in Alzheimer’s disease (AD) [6], both at the symptomatic and presymptomatic stages of the disease. Previous studies have implicated links between AxD and Alzheimer’s disease, both in terms of oxidative stress [3] and transcriptomic profiles of gene expression [4].

In this study, in addition to GFAP, we sought to determine whether these novel blood biomarkers might prove useful in AxD. Here, we report that GFAP is elevated in plasma of all age groups afflicted by AxD, including those with adult onset. NfL and p-tau are also elevated, but to a much lesser extent than GFAP. In contrast, the levels of Aβ40 and Aβ42 are not altered in AxD.

## Methods

### Participants

Blood samples from AxD individuals (*n* = 49) and controls (*n* = 31) were the same as those analyzed previously in Jany et al. [7]. Briefly, AxD participation required genetic confirmation of the diagnosis by sequencing of the *GFAP* gene. This cohort contained those with neonatal (*n* = 3), infantile (*n* = 21), juvenile (*n* = 12), and adult onsets (*n* = 13), with 27 different variants distributed throughout the rod and tail domains of GFAP (see Supplemental Data for details of variants). Controls were unaffected healthy adults (≥ 18 years) of both sexes. Fresh samples of venous blood were collected into lavender-topped tubes that contained K_2_-EDTA as anticoagulant. The samples were centrifuged within 60 min of collection at 2500 *g* for 15 min at room temperature. The supernatant was immediately placed in a polypropylene tube and stored either on dry ice for shipping or at − 20 °C until shipping could be arranged. Upon arrival at the central laboratory, the samples were thawed, divided into aliquots, and stored at − 80 °C until further analysis. Three blood samples were collected as serum rather than plasma and were considered non-standard.

### Blood biomarker measurements

All plasma biomarker measurements were performed using single molecule array (Simoa) technology on an HD-X platform at the University of Gothenburg, Sweden, blinded to participant information. Plasma GFAP, NfL, Aβ42, and Aβ40 concentrations were measured using the commercial Neurology 4-plex E kit (#502,334, Quanterix, Billerica). Plasma p-tau181 and p-tau231 concentrations were measured using in-house Simoa assays developed at the University of Gothenburg [1, 9]. All measurements were done in singlicates on samples having undergone two free-thaw cycles and performed on one occasion using one batch of reagents. Intra-assay coefficients of variation on all biomarkers were < 15% derived from the internal control samples measured in duplicate on each analytical run. The three patient samples collected as serum were included in the analysis. Removing them from the study did not affect the results.

### Statistical analysis

Data normality was determined by the D’Agostino-Pearson test, and statistical evaluation was performed on log_10_-transformed data. All data analysis reported has been performed on log_10_-transformed data, but the untransformed values are shown in descriptive tables and figures. A one-way ANOVA (Kruskal–Wallis) was performed to compare biomarker levels across groups adjusted for multiple comparisons. Correlations between the age of onset, age at collection, and between biomarkers were performed by Spearman’s rank correlation. Statistical analysis was performed using IBM SPSS Statistics, version 25 (Armonk, NY, USA), and graphical representation was performed in Graph Pad Prism.

### Ethics

Informed consents for studies of blood were obtained following protocols approved by the Institutional Review Board at the University of Wisconsin-Madison, and in accordance with the ethical standards from the 1964 Declaration of Helsinki and its later amendments.

## Results

The demographics of the cohort are shown in Table 1. The mean age of the control group was 34.6 years, whereas the mean ages of AxD patients (at the time when their samples were collected, though grouped by age of onset) were as follows: neonatal = 2.2, infantile = 6.6, juvenile = 19.9, and adult = 43.5 (all in years). The AxD group as a whole was balanced for sex, whereas the control group had a bias toward female participants.

**Fig. 1:**
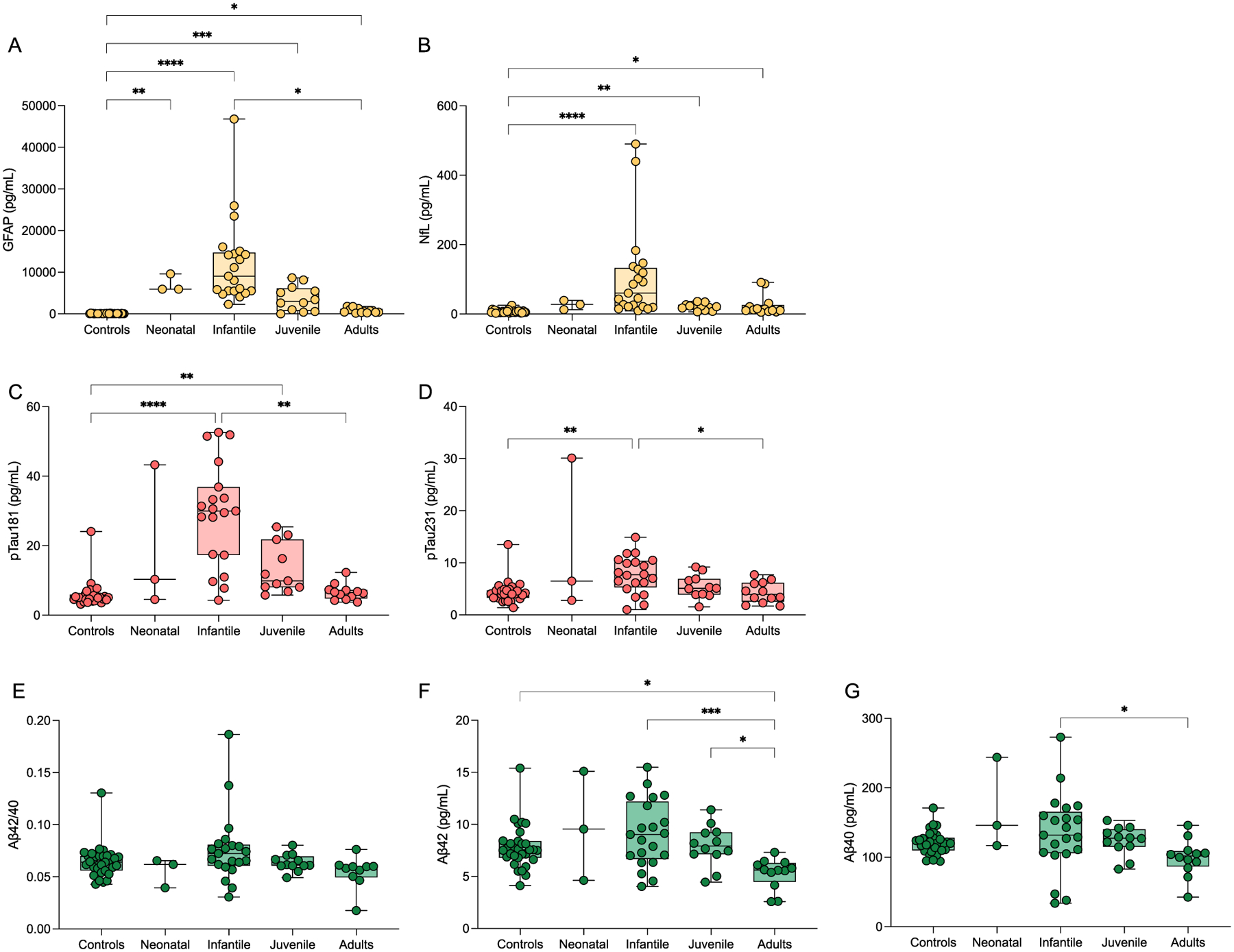
Box and whisker plots demonstrating the concentrations of blood GFAP (**A**), NfL (**B**), pTau181 (**C**), pTau231 (**D**), Aβ42/40 (**E**), Aβ42 (**F**), and Aβ40 (**G**) in controls and AxD patients separated by age of onset. The age of onset classification boundaries are as in Jany et al. (2015) with the addition of the neonatal form (years): neonatal (0–0.08), infantile (0.08–2), juvenile (> 2–13), adult (> 13). *P < 0.05, **P < 0.01, ***P < 0.001, ****P < 0.0001

Plasma levels of GFAP (Fig. 1a) were significantly increased in neonatal AxD (mean (SD); 7164 pg/mL (2126), *P* = 0.008), infantile AxD (12,191 pg/mL (10,158), *P* < 0.0001), and juvenile AxD (3705 pg/mL (3018), *P* < 0.001) compared with controls (70.6 pg/mL (38.1)). There was also a significant increase in adult AxD GFAP levels (774.1 pg/mL (550), *P* = 0.024). Plasma NfL (Fig. 1b) was increased in infantile AxD (106 pg/mL (130), *P* < 0.0001), juvenile AxD (21.1 pg/mL (9.6), *P* = 0.003), and adult AxD (24.6 pg/mL (29.6), *P* = 0.032) compared with controls (7.1 pg/mL (4.4)). The apparent change in NfL for neonatal AxD (26.37 pg/mL (13.4)) was non-significant. (Log scales for GFAP and NfL are shown in Supplemental Fig. 1).

Plasma p-tau181 (Fig. 1c) and p-tau231 (Fig. 1d) demonstrated a similar pattern, with highest levels observed in infantile AxD (p-tau181, 28.9 pg/mL (15.1), *P* < 0.0001; p-tau231, 7.7 pg/mL (3.4), *P* = 0.001) compared with controls (p-tau181, 5.9 pg/mL (3.6); p-tau231, 4.3 pg/mL (2.0)). However, in contrast, p-tau181 was significantly increased in juvenile AxD (13.3 pg/mL (7.1), *P* = 0.003) compared to controls, which was not observed for p-tau231. Plasma Aβ42/40 (Fig. 1e) was unchanged across all groups, although Aβ42 (Fig. 1f) and Aβ40 (Fig. 1g) peptides were lowest for the adult AxD groups (Aβ42, 5.3 pg/mL (1.4), Aβ40, 99.2 pg/mL (26.3)).

**Fig. 2:**
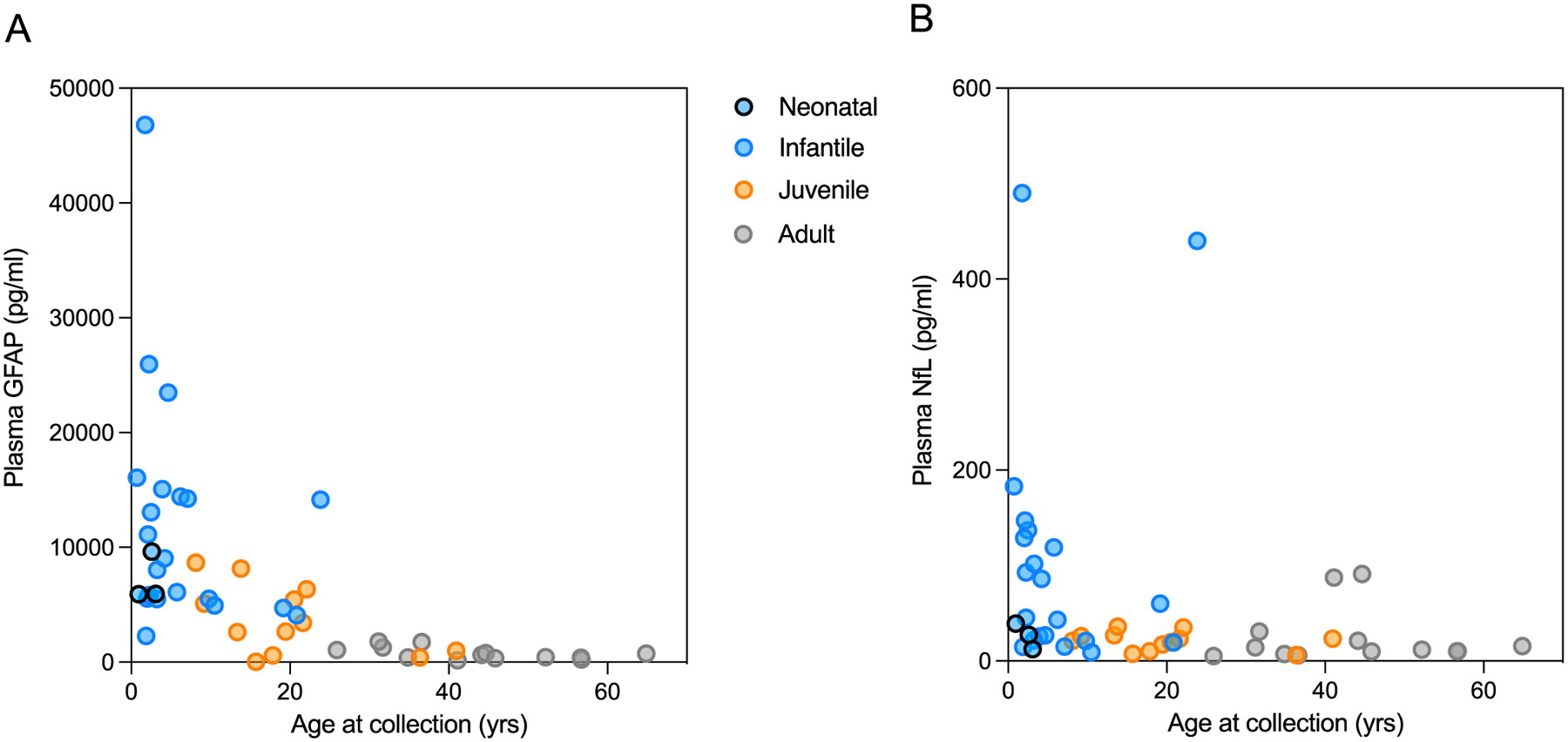
Correlation plots demonstrating the concentrations of GFAP (**A**) and NfL (**B**) as a function of age at sample collection in AxD patients. AxD patients are colour coded by the age of onset as neonatal (blue with black border), infantile (blue), juvenile (orange), and adult (grey)

There was a significant overall correlation between plasma GFAP and NfL (r = 0.533, *P* < 0.001), which had a slightly stronger association in AxD patients (r = 0.663, *P* < 0.001). All blood biomarkers had a negative association with age at collection in the AxD group (GFAP, r = − 0.766, *P* < 0.0001 (Fig. 2A); NfL, r = − 0.528, *P* < 0.0001 (Fig. 2B); p-tau181, r = − 0.613, *P* < 0.0001; p-tau231, r = − 0.381, *P* = 0.009; Aβ40, r = − 0.389, *P* = 0.006; Aβ42, r = − 0.395, *P* = 0.005). With respect to GFAP, it is interesting to note that the age at collection appeared to be a more important factor than the age of onset.

## Discussion

We found significant changes in the levels of GFAP, NfL, and p-tau in the blood of individuals with AxD, especially those with infantile-onset AxD. This is the first study to show changes in plasma NfL, a biomarker associated with the intensity of neurodegeneration, in AxD. The higher levels of NfL in infantile-onset AxD are consistent with this group having shorter survival time and clinical manifestations of encephalopathy and epilepsy. Similarly, ours is the first study to show changes in p-tau, specifically p-tau181, in AxD. This biomarker is thought to be a specific biomarker for AD pathology [10], but AD is an unlikely scenario in the AxD patient group. One can speculate that p-tau181 is reflecting intensity of brain injury in AxD; however, the lack of correlation between NfL and p-tau181 in the juvenile AxD group suggests another mechanism, such as blood–brain barrier dysfunction. For GFAP, the results reported here differ from those reported in an earlier study [7], where adult-onset AxD individuals were indistinguishable from controls. In Jany et al. [7], however, GFAP quantitation was performed using a less sensitive sandwich ELISA in which 41% of the controls were not measurable. Using the Simoa platform, with an improved analytical sensitivity and wider dynamic range, the same samples now show that adult-onset patients have blood values that, as a group, are tenfold higher than controls.

Reactive astrocytes are a prominent feature in many of the leukodystrophies, and whether changes in CSF and/or blood levels of GFAP suggest utility as a biomarker for these conditions is just beginning as a topic for investigation. Recently, Beerepoot and colleagues [2] showed elevations in both GFAP and NfL in metachromatic leukodystrophy, although the degree of increase for GFAP was less than that seen in AxD. Of most significance was their finding that the degrees of elevation could distinguish slow vs. rapid rates of progression in the children with onsets before the age of 6 years. Changes in CSF and blood levels of GFAP and NfL have also been observed in X-linked adrenoleukodystrophy [18].

Our study has several limitations. With regard to GFAP, Petzold [14] pointed out that none of the existing assays addresses potential differences in the expression of isoforms, post-translational modifications, cleavage products, or the hook effect as seen with other protein aggregates. In addition, without careful epitope mapping, the values for patients may be impacted by whether their individual variant affects assay performance, especially in those individuals with major deletions. Second, our study had imperfect age-matching between patients and controls (the latter limited by IRB requirements to be ≥18 years). However, prior studies on the same analytes with similar methods in younger controls suggest that the current interpretations for the AxD data set are correct [16, 17]. For p-tau, concentrations are relatively high in newborns but become indistinguishable from adult levels in children over the age of 1 year, speaking against age being a major confounder when interpreting our results [12]. Third, we possessed limited clinical information on the AxD patients, thus preventing utilization of any classification system other than age of onset. Interesting differences may emerge when these biomarkers are evaluated in the context of the alternative systems proposed by Prust et al. [15] and Yoshida et al. [19]. Fourth, our results represent, for each patient, only a single point in time, and longitudinal studies that follow change over time will be extremely informative.

In conclusion, there is a need for easily obtainable biomarkers that can assist in monitoring disease progression and treatment response in AxD. Our novel results show that in addition to GFAP, blood biomarkers of neural injury (NfL) and tau (p-tau181) are changed in AxD, particularly those with infantile onset, and should be further examined in the wider context of AxD.

## Supplementary Material

Supplementary File 1

Supplementary File 2

## Figures and Tables

**Table 1:** Demographics of the study cohort (ages in years)

	Number (m/f)	Age (mean)	Age (SD)	Age (range)
Controls	31 (9/22)	34.6	10.9	19–57
AxD (total)	49 (24/25)			
*Neonatal*	3	2.2	1.1	0.96–3.07
*Infantile*	21	6.6	6.7	0.72–23.9
*Juvenile*	12	19.9	9.9	8.14–41.0
*Adult*	13	43.6	11.6	13.8–64.9
