# Supplementary material for: Plasma concentrations of glial fibrillary acidic protein, neurofilament light, and tau in Alexander disease
Source: Neurol Sci. 2024 Apr 1;45(9):4513–8. doi: 10.1007/s10072-024-07495-8 (PMC11305938; doi:10.1007/s10072-024-07495-8)
Supplement: Supplementary file 1 — Supplementary file1 (DOCX 16 KB) [file 10072_2024_7495_MOESM1_ESM.docx]

*Supplemental Data*

**Number of participants with each GFAP variant shown in parentheses**

Arg70Gln (1)

Asn77Ser (3, one also with Ser152Leu)

Arg79Cys (3)

Arg79Gly (1)

Arg79His (3)

Arg79Leu (1)

Arg88Cys (6)

Arg105W (1)

Leu123Pro (1)

Glu207Gln (1)

Leu231His (2)

Arg239Cys (5)

Arg239His (2)

Arg239Pro (2)

Ser247Pro (1)

Arg258Pro (1)

Arg270-Ala272del (1)

Gln290Glu (1)

Glu362Gln (1)

Glu371Gln (1)

Glu373Ala (1)

Ser398Phe (1)

Ser398Tyr (1)

Met415Ile (2, one also with Asp157Asn)

Arg416W (4)

Asp417Ala (1)

Gln426Leu (1)
