# Supplementary figures and images for: Plasma concentrations of glial fibrillary acidic protein, neurofilament light, and tau in Alexander disease
Source: Neurol Sci. 2024 Apr 1;45(9):4513–8. doi: 10.1007/s10072-024-07495-8 (PMC11305938; doi:10.1007/s10072-024-07495-8)

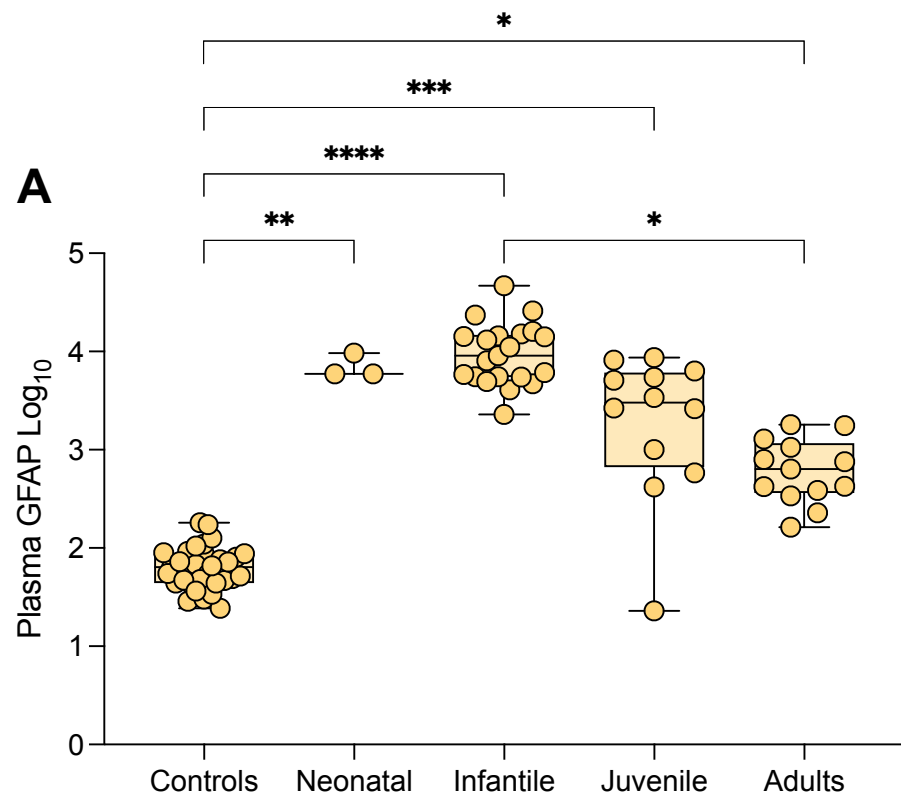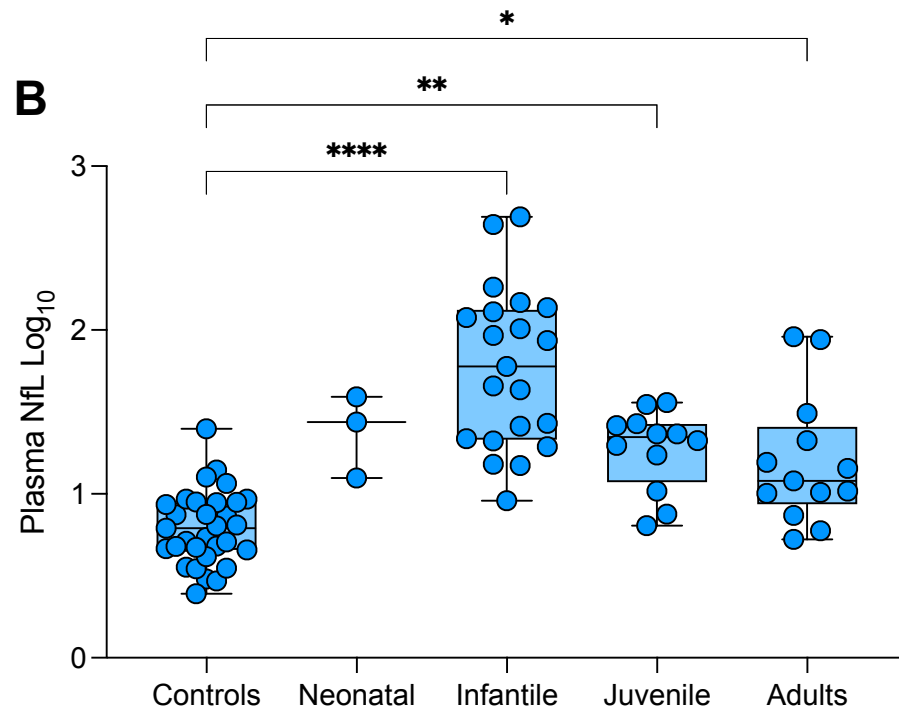

Supplement: Supplementary file 2 — Supplementary file2 (PDF 69 KB) [file 10072_2024_7495_MOESM2_ESM.pdf]
